# Effects of Grape Seed Extract Supplementation on Hemodynamic Response and Vascular Endothelial Function in Postmenopausal Women

**Published:** 2019-09

**Authors:** Dae-Sung LEE, Jooyoung KIM

**Affiliations:** 1. Department of Physical Education, Kyunghee University, Suwon, Korea; 2. Department of Anatomy, School of Medicine, Kyungpook National University, Daegu, Korea

## Dear Editor-in-Chief

Menopause is defined as the complete loss of female fertility after the cessation of both menstruation and ovarian function (1). These changes increase the incidence of chronic diseases and, particularly, elevate the risk of cardiovascular diseases in postmenopausal women (2). Supplementation of polyphenols can serve as an alternative method to stimulate estrogen secretion (3, 4). Grape seeds contain various polyphenols (5) at a concentration of approximately 2178.8 mg/g gallic acid equivalent (GAE), which is considerably higher than that observed in grape skin (374.6 mg/g GAE) and grape leaves (351.6 mg/g GAE) (6). Grape seed extract (GSE) supplementation is known to induce estrogen synthesis by normalizing estrogen receptor deficiency (3).

These findings indicate that GSE supplementation has potentially positive effects on changes in the hemodynamic response and vascular endothelial function in postmenopausal women. However, to date, only a few studies have investigated the effect of GSE supplementation in postmenopausal women and the associated changes in hemodynamic response and vascular endothelial function.

Therefore, in the present study, we evaluated the effects of GSE supplementation on hemodynamic response and vascular endothelial function in postmenopausal women.

Eleven postmenopausal women (age: 53.6 ± 0.8 years; height: 156.8 ± 1.8 cm; body weight: 55.7 ± 2.4 kg; body fat: 29.7% ± 1.2%) were enrolled in the study.

All participants signed informed consent form and the study was approved by the Kyunghee University Institutional Review Board.

In this randomized, double-blind, crossover trial, the participants were subjected to multiple rounds of GSE and placebo supplementation experiments (300 mg of GSE or placebo per day for 4 weeks, with a 2-week washout period between doses). To assess the hemodynamic response, heart rate (HR), stroke volume (SV), cardiac output (CO), total vascular conductance (TVC), systolic blood pressure (SBP), diastolic blood pressure (DBP), and mean arterial pressure (MAP) were measured. Vascular endothelial function was measured using flow-mediated dilatation (FMD). The measured variables were analyzed through repeated measure analysis of variance. The level of significance was set at *P* < 0.05.

GSE supplementation reduced the SBP (*P* < 0.05), but increased the FMD (*P* < 0.001) in postmenopausal women (Table 1). However, there were no significant differences in the HR, SV, CO, TVC, DBP, and MAP between the groups. Furthermore, no significant differences were observed in any of the variables in the placebo group (Table 2).

**Table 1:** Effects of grape seed extract (GSE) supplementation on hemodynamic response in postmenopausal women

***Variable***	***Trials***	***Pre***	***Post***	***P***	
HR (beats/min)	Polyphenol	56.5 ± 1.1	57.5 ± 1.5	Time	0.085
				Trial	0.890
	Placebo	56.6 ± 1.7	58.0 ± 1.0		
				Interaction	0.735
SV (ml)	Polyphenol	59.8 ± 2.0	60.1 ± 2.5	Time	0.615
				Trial	0.894
	Placebo	60.0 ± 2.2	59.0 ± 1.8		
				Interaction	0.350
CO (l/min)	Polyphenol	3.3 ± 0.1	3.4 ± 0.1	Time	0.316
				Trial	0.982
	Placebo	3.4 ± 0.1	3.4 ± 0.1		
				Interaction	0.905
TVC (ml/min/mmHg)	Polyphenol	35.4 ± 0.8	37.7 ± 1.3	Time	0.154
				Trial	0.691
	Placebo	35.9 ± 1.8	35.9 ± 1.0		
				Interaction	0.148
SBP (mmHg)	Polyphenol	121.0 ± 3.6	117.2 ± 2.6	Time	0.880
				Trial	0.745
	Placebo	118.2 ± 3.4	122.6 ± 2.7		
				Interaction	0.049*
DBP (mmHg)	Polyphenol	82.8 ± 2.5	78.2 ± 1.1	Time	0.111
				Trial	0.303
	Placebo	83.9 ± 2.6	82.1 ± 2.2		
				Interaction	0.486
MAP (mmHg)	Polyphenol	95.5 ± 2.8	91.2 ± 1.4	Time	0.275
				Trial	0.451
	Placebo	95.4 ± 2.7	95.6 ± 2.3		
				Interaction	0.223

Value are expressed as mean ± standard error, HR: heart rate, SV: stroke volume, CO: cardiac output, TVC: total vascular conductance, SBP: systolic blood pressure, DBP: diastolic blood pressure, MAP: mean arterial pressure

* *P*<0.05; tested by repeated measure analysis of variance

**Table 2:** Effects of grape seed extract (GSE) supplementation on vascular endothelial function in postmenopausal women

***Variable***	***Trials***	***Pre***	***Post***	***P***	
FMD (%)	GSE	10.2 ± 1.5	15.7 ± 1.9	Time	0.822
				Trial	0.169
	Placebo	19.1 ± 3.1	14.5 ± 2.3		
				Interaction	0.018*

Values are expressed as mean ± standard error, FMD: flow mediation dilatation

* *P*<0.05; tested by repeated measure analysis of variance

These results showed that GSE supplementation has a positive effect on blood pressure and vascular endothelial function in postmenopausal women. Thus, it can be used as a nutritional intervention to lower the risk of cardiovascular diseases in postmenopausal women.
